# Enthesitis Related Arthritis in a Longitudinal Southeast Asian Registry: High Prevalence of HLA-B27, Different Sacroiliitis Risk Factors and Less Common Drug-Free Remission

**DOI:** 10.3390/jcm10040568

**Published:** 2021-02-03

**Authors:** Thaschawee Arkachaisri, Kai Liang Teh, Yun Xin Book, Sook Fun Hoh, Xiaocong Gao, Lena Das

**Affiliations:** 1 Rheumatology and Immunology Service, Department of Pediatric Subspecialties, KK Women’s and Children’s Hospital, Singapore 229899, Singapore; teh.kai.liang@singhealth.com.sg (K.L.T.); book.yun.xin@kkh.com.sg (Y.X.B.); lena.das@kkh.com.sg (L.D.); 2 Duke-NUS Medical School, Pediatric Academic Clinical Program, Singapore 169857, Singapore; 3 Division of Nursing, KK Women’s and Children’s Hospital, Singapore 229899, Singapore; hoh.sook.fun@kkh.com.sg (S.F.H.); gao.xiaocong@kkh.com.sg (X.G.)

**Keywords:** juvenile idiopathic arthritis, enthesitis-related arthritis, Southeast Asia, predictors, Singapore, treatment

## Abstract

**Objective**. To describe the clinical characteristics, predictors and treatment of children with Enthesitis Related Arthritis (ERA) in a Singapore longitudinal cohort over 11 years. **Methods**. ERA patients were recruited from our registry (2009–2019). Nonparametric descriptive statistics including median (interquartile range, IQR) were used to describe data. Kaplan–Meier survival and logistic/Cox regression analyses were used to estimate the probabilities and determine predictors of clinical variables, respectively. The significance level was set at <0.05. **Results**. One hundred and forty-six ERA patients (87% male, 82% Chinese) were included. Median onset age was 11.9 years (IQR 9.4–14.0) and median disease duration was 4.9 years (IQR 2.6–8.3). Family history of Human Leukocyte Antigen (HLA)-B27 associated diseases was positive in 7.5%. Acute uveitis occurred in 3.4%. Oligoarthritis was present in 89.7%. Hip, knee and ankle joints were among the most common joints involved. One-fourth had enthesitis at diagnosis (Achilles tendon entheses, 82.9%). Sacroiliitis occurred in 61%. Probabilities of sacroiliitis development were 0.364, 0.448 and 0.578 at 1, 2 and 5 years after onset, respectively. Negative HLA-B27, female, older age at onset and hip arthritis at diagnosis were associated with shorter time for sacroiliitis development (*p* = 0.001–0.049). Methotrexate (MTX) remained the most common disease modifying anti-rheumatic drug (DMARD) used (77.4%). However, 77.9% required anti-TNF (aTNF) therapy secondary to MTX failure. Among MTX-treated sacroiliitis patients, 85.3% failed, requiring aTNF, as compared to 63.2%patients without axial disease. Longer duration to diagnosis (*p* = 0.038) and MTX use (*p* = 0.007) predicted aTNF therapy. None had joint deformity. **Conclusions**. This study underscores differences in ERA clinical characteristics, predictors and treatment responses. Our ERA population had many unique findings but good functional outcomes.

## 1. Introduction

Enthesitis Related Arthritis (ERA) is a subtype of Juvenile Idiopathic Arthritis (JIA) with unique characteristics, including male predominance with later onset, Human Leukocyte Antigen (HLA)-B27 association as well as enthesitis and axial skeleton in addition to peripheral joint involvements, which differentiates it from other JIA categories. ERA accounts for 5–15% of JIA reported in the Western, Middle Eastern and some Asian cohorts [1]. Interestingly, it is significantly higher, at least one-third of JIA, in India, Singapore and Taiwan, compared to other Asian cohorts [2,3,4,5,6,7]. Patients with ERA tend to have more chronic disease and poorer physical outcomes compared with oligoarticular and polyarticular JIA in long-term studies [4,8,9].

In a multi-ethnic Canadian cohort, more ERA prevalence was observed in those of Asian descent [10]. However, even among Asian JIA cohorts, the frequencies of ERA vary, underscoring the crucial role of genetic factors in the etiopathogenesis and phenotype of the disease (see below) [10,11]. Due to its low prevalence in the West, reports on ERA clinical characteristics, treatment and outcomes are scarce compared to other JIA categories. Even in Asia with higher ERA prevalence, there is only one study with detailed clinical features and outcomes reported from Taiwan [4]. This limitation not only makes comparative study difficult and inaccurate given the lack of information for interested researchers but also makes it harder for physicians caring for patients with ERA to counsel patients and family members when the disease is diagnosed. Moreover, since the prevalence of ERA in Asia alone is diverse, one would wonder if the phenotype and/or natural history of this particular JIA category would be identical among ethnic groups, especially among these three higher ERA prevalence countries.

Since ERA is the most common JIA category in Singapore, we aimed to describe the clinical characteristics, natural history, treatment and functional outcome of our ERA inception cohort [6,12]. We also aimed to identify the predictors of clinical variables and compare them with reports elsewhere.

## 2. Materials and Methods

### 2.1. Study Population

Patients diagnosed with ERA according to the International League of Associations for Rheumatology (ILAR) classification criteria, with at least 2 follow-up visits and 6 months disease duration, were recruited from our REgistry for Childhood Onset Rheumatic Diseases (RECORD), an on-going web-based registry based at KK Women’s and Children’s Hospital, Singapore, from January 2009 to August 2020 [13]. Demographic and clinical data along with treatment, physician’s global assessment of disease activity (PGA), laboratory tests and imaging studies were collected. These observational data were collected retrospectively for patients diagnosed prior to 2009 and then prospectively after enrollment. SingHealth Centralised Institutional Review Board approved the study (CIRB 2019/2274, 2019/2961).

### 2.2. Definitions

Active joint count was defined as the number of swollen joints or joints with limited range of motion accompanied by pain on motion and/or tenderness. Enthesitis was defined as tenderness over the entheseal site by palpation and/or demonstrated by MRI. Active sacroiliitis was defined by the presence of bone marrow edema and contrast enhancement at the sacroiliac joint (SIJ) using The Assessment of SpondyloArthritis international Society (ASAS) criteria [14]. The MRI of SIJ was not routinely obtained unless the patients were symptomatic or had HLA-B27-positive with restricted modified Schober’s test. All patients underwent regular uveitis screening by ophthalmologists. Outcome statuses at last visit were defined according to the criteria for inactive disease and remissions of JIA proposed by Wallace, et al., with modification [14,15]. Additional criteria were applied including no enthesitis and no active sacroiliitis on MRI [14].

### 2.3. Treatment Approach

We routinely started ERA patients with nonsteroidal anti-inflammatory drugs (NSAIDs) as our first line of treatment for both peripheral and axial diseases. The conventional disease modifying anti-rheumatic drugs (cDMARDs) including sulfasalazine (SSZ), methotrexate (MTX) and leflunomide were added as second line therapy. Biological DMARDs (bDAMRDs) were added only after inadequate response to or failure of cDMARDs occurred. Intra-articular steroid injection or systemic corticosteroids therapy were used as adjunct and bridging therapy, respectively. Despite the axial involvement, it was our local prerequisite to demonstrate MTX failure prior to bDMARDs addition for third-party financial assistance.

### 2.4. Statistical Analysis

Nonparametric analyses were used to describe data and are shown as median (interquartile range, IQR or range) for continuous variables and percentages for categorical variables. Chi-squared or Fisher’s exact and Mann–Whitney U tests were applied to compare differences between groups where appropriate. Survival tables and Kaplan–Meier time-to-event curves were constructed from the date of onset to determine cumulative probability of sacroiliitis development and from the date of diagnosis to determine cumulative probabilities of treatments (MTX, anti-TNF (aTNF)) initiation. Predictors of sacroiliitis development were examined using logistic regression. Variables that gave a *p* < 0.1 in the univariate logistic regression were entered into the multivariate logistic regression by a backward conditional method and confirmed with a forward conditional method yielding odd ratios (ORs) and 95% confidence intervals (95%CI) for predictor variables. The same procedure was applied to Cox regressions to investigate the effects of variables upon the time to sacroiliitis development. All analyses were performed using SPSS, version 23.0 (IBM Corp., Armonk, NY, USA) with statistical significance set at *p* < 0.05.

## 3. Results

### 3.1. Patients Characteristics

Between January 2009 to August 2020, 473 patients were diagnosed with JIA. One hundred and fifty-two patients fulfilling the International League of Associations for Rheumatology (ILAR) classification criteria for ERA were identified and 146 followed for at least 6 months were included, Table 1. Eighty-eight patients enrolled prior to 2015—whose data were partially reported elsewhere-were included [6,16]. Chinese (81.5%), male (87.0%) and HLA-B27-positive (82.2%) patients composed the majority. The median age at onset was 11.9 years (IQR 9.4–14.0) and median duration of disease was 4.9 years (IQR 2.6–8.3). The median duration from onset of first symptoms to diagnosis (lag period) was 2.9 months (IQR 1.2–7.1). Uveitis occurred only in 3.4%. Table 2 reveals each of the ILAR criteria for ERA diagnosis fulfillment. All except one patient fulfilled the classification criteria at their first presentation. Only 18.5% presented with peripheral arthritis and enthesitis, 7.5% had family history of HLA-B27 associated diseases in the first degree relative and majority (84.9%) were boys over six years old at onset. There was no acute uveitis at onset.

### 3.2. HLA-B27

Table 1 illustrates the clinical characteristics of patients with and without HLA-B27. The two groups were largely similar in clinical characteristics except for more Indian descent (*p* = 0.019) and more sacroiliitis either at diagnosis (*p* < 0.001) or ever (*p* = 0.027) in patients with negative HLA-B27. More SSZ was used in the positive HLA-B27 group (*p* < 0.001).

### 3.3. Peripheral Joint Distribution and Enthesitis

**Figure 1:**
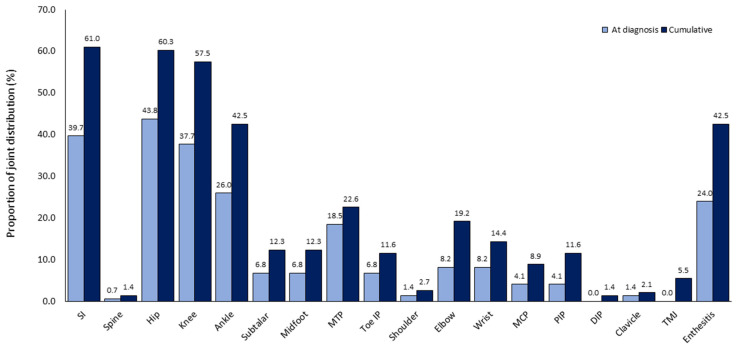
Proportion of affected joint distribution (%) at diagnosis (gray) and during the course of the disease (cumulative, black). SI = sacroiliac joint, MTP = metatarsophalangeal joint, IP = interphalangeal joint, MCP = metacarpophalangeal joint, PIP = proximal interphalangeal joint, DIP = distal interphalangeal joint, TMJ—temporomandibular joint.

The majority of patients presented with oligoarthritis (89.7%). The median number of active joints at diagnosis was 2 (IQR 1–3). Hip (43.8% vs. 60.3%), knee (37.7% vs. 57.5%) and ankle (26.0% vs. 42.5%) were among the common joints involved at diagnosis and along the course of the disease, respectively (Figure 1). Enthesitis developed in 62 patients and the Achilles tendon enthesis remained the most commonly involved (88.7%). Among patients with enthesitis, 35 had enthesitis at diagnosis for which Achilles tendon enthesis was the most prevalent (82.9%). Only 8 patients presented with enthesitis without arthritis at diagnosis.

### 3.4. Axial Involvement

Eighty-nine patients had sacroiliitis (84.3% male) of which 58 presented at diagnosis, with bilateral SIJ involvement in 34 patients. Only 62 (69.7%) patients were symptomatic. Median age at first sacroiliitis was 13.7 years (IQR 10.8–15.8) and was not different between genders (*p* = 0.187). ERA patients with sacroiliitis had HLA-B27 positive in 76.4% as compared with 91.2% of those without sacroiliitis during the study period (Supplemental Table S1). The probabilities of sacroiliitis development are shown in Table 3. Considering only ERA patients, HLA-B27 decreased (OR 0.311, 95%CI 0.106–0.913, *p* = 0.034) and hip arthritis at diagnosis increased (OR 3.941, 95%CI 1.869–8.311, *p* < 0.001) the risk of sacroiliitis during the study period (Supplemental Table S2). Furthermore, boys (HR 0.508, 95%CI 0.282–0.914, *p* = 0.024) and HLA-B27 (HR 0.0452, 95%CI 0.272–0.752, *p* = 0.024) had longer but older age at onset (HR 1.076, 95%CI 1.000–1.156, *p* = 0.049) and hip arthritis at diagnosis (HR 2.128, 95%CI 1.390–3.257, *p* = 0.001) had shorter time to develop sacroiliitis (Supplemental Table S3). Knee arthritis at diagnosis (OR 0.237, 95%CI 0.080–0.705, *p* = 0.010) and corticosteroid use (OR 0.355, 95%CI 0.128–0.982, *p* = 0.046) decreased the risk of early sacroiliitis (within the first year after onset). Furthermore, patients with early sacroiliitis tended to seek medical attention earlier (shorter lag period, *p* = 0.002) (Supplemental Table S2).

### 3.5. Treatment

Almost all ERA patients had NSAIDs (97.3%) and one-half of the patients required corticosteroids (Table 1). Only 29.5% had intra-articular steroids injection. The majority of the patients had SSZ (78.8%). All 12 patients taking SSZ alone without further therapy escalation had peripheral joint diseases without axial involvement. Methotrexate was prescribed to 113 patients. Anti-TNF (aTNF) was the most commonly used biologic, 72.6%. Sixty-three percent of peripheral arthritis compared with 85.3% of sacroiliitis patients failed MTX, requiring aTNF therapy. Probabilities of taking MTX were 0.649, 0.716, 0.814 and aTNF were 0.461, 0.581, 0.775 at 1, 2 and 5 years after diagnosis, respectively (Table 3). Longer duration from onset to diagnosis associated with aTNF use (OR 1.087, 95%CI 1.005–1.176, *p* = 0.038). As expected, MTX use predicted aTNF use by 3.2 times (95%CI 1.379–7.539, *p* = 0.007) (Supplemental Table S2).

### 3.6. Outcomes

**Figure 2:**
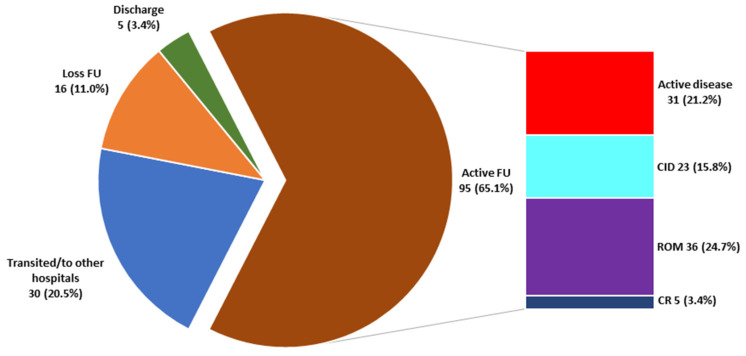
Number and proportion of enthesitis-related arthritis patient status at their last visit (*n* = 146). FU = follow-up, CID = clinical inactive disease, ROM = remission on medication, CR = clinical remission off medication.

With a median follow-up period of 4.2 years (range 0.5–14.7) and median age at last follow-up of 17.1 years (range 8.4–20), 30 patients were transited to adult rheumatologists (85% remained on medication but all had inactive disease), 5 patients were discharged, while 16 patients were lost to follow-up, leaving 95 patients remaining under our care with different disease status as shown in Figure 2. Of all 146 ERA patients at their last visit, only twelve (8.2%) patients could discontinue all their medications for at least 12 months (clinical remission off medication, CR) while 52 patients could maintain their inactive disease state on medications for at least 6 months (remission on medication, ROM). One-third of the patients suffered active disease while 33 patients achieved clinical inactive disease, CID. No patient had joint deformity or became disabled. HLA-B27 did not affect the outcome status at the last visit as 81.6% of the active vs. 82.5% of the non-active (CID + ROM + CR) ERA patients had HLA-B27 (*p* = 0.900).

## 4. Discussion

This is the first Southeast Asian study that attempted to describe the clinical characteristics, treatment and functional outcomes of ERA patients followed in a large pediatric rheumatology center in Singapore where ERA category was the most common and contributed one-third of the total JIA population, similar to reports from Taiwan and India [4,5,6,11]. The study contains the largest monocentric ERA cohort published to date, which is only second to a US multicentered study reported by Gmuca et al. in 2017 [17]. Compared with other ERA cohorts reported, we observed significant differences in disease phenotype, natural history and functional outcomes, underscoring the crucial roles of ethnicity, genetic and/or environmental factors in the ERA immunopathogenesis, and in shaping the clinical phenotype, therapeutic approach, response to treatment and overall outcomes.

### 4.1. Demographic and Clinical Characteristics

In this cohort, Chinese descent composed the majority, and Malays and Indians were underrepresented compared with the racial distribution of Singapore’s population. As expected, males made up the majority (87%), with median age at onset 11.9 years, similar to reports elsewhere (Table 4) [2,4,7,11,17,18,19]. Median duration from onset to diagnosis (lag period) was 2.9 months, which was much shorter than that of Canadian (Research in Arthritis in Canadian, Emphasizing Outcomes (ReACCh-Out), 5.9 months) and French combined ERA/juvenile spondyloarthritis (jSpA) (mean 1.2 years) cohorts [19,20]. Raising JIA awareness in Singapore around the time of the establishment of our pediatric rheumatology clinical program in 2009 and easier tertiary care access (Singapore distance: east–west 50 km, north–south 27 km) might have played critical roles in shortening our lag period. All but one patient fulfilled the ILAR ERA classification criteria at first presentation. Peripheral arthritis was present in 95% of our patients, similar to other cohorts, 92–97% [4,11,17]. However, enthesitis at diagnosis was observed in only 24%, which was much less than 97% from Taiwanese, 96% from Indian and 75% from the US cohorts [4,11,17]. Moreover, males over 6 years old (85%) and the presence of HLA-B27 (82%) were among the most common ILAR criteria fulfilled, similar to the Taiwanese cohort. Surprisingly, only 8% of our ERA patients had a family history of HLA-B27-related diseases, which was less than reports elsewhere, despite our high HLA-B27 prevalence (Table 4) [2,4,11,17,19]. The explanation remains unclear. Acute anterior uveitis was uncommon either at diagnosis (none) or later in the course of the disease (3.4%), compared with reports from other Asian and Western cohorts (Table 4) [2,4,7,11,17,18,19]. The low uveitis incidence paralleled our low incidence of uveitis in other JIA categories, suggesting that there may be local genetic factors protecting against this extra-articular complication [6,12].

### 4.2. HLA-B27

Higher prevalence of HLA-B27 was observed in our (82%), Taiwanese (92%) and Indian (79%) cohorts compared to other Asian and Western cohorts [2,4,7,11,17,18,19]. This Major Histocompatibility Complex (MHC) Class I has been studied well in many adult ankylosing spondylitis (AS) cohorts and was shown to be associated with more male, younger age at onset, higher family aggregation of disease, less prevalence of peripheral arthritis, more hip arthritis, enthesitis and sacroiliitis prevalence and seems to have higher frequency of acute anterior uveitis in AS patients [21]. A recent US multicentered study found that children who were HLA-B27-positive were older and more males with higher sacroiliitis and active peripheral joint counts [17]. Our ERA children with HLA-B27 tended to be older with a shorter lag period, more family history, more acute uveitis, more active joint counts and higher inflammatory marker levels (Erythrocyte Sedimentation Rate (ESR), C-Reactive Protein (CRP)), but these did not reach statistical significance. The low number of patients may partly contribute to these results. Surprisingly, we observed less sacroiliitis and hip arthritis either at diagnosis or later but similar male prevalence in our children with ERA and HLA-B27-positive compared with the HLA-B27-negative group (see below). Furthermore, enthesitis tended to be less at presentation but similar frequency later on in both groups was noted.

### 4.3. Peripheral Joint and Axial Involvement

The majority of our ERA patients presented with oligoarthritis (90%) similar to other cohort observations [4,17]. However, hip was the most common joint involved both at diagnosis (44%) and later (60%), followed by knee (38% and 58%) and ankle (26% and 43%) joints compared with the American, Taiwanese, Chinese and Canadian cohorts where knee arthritis was the most common joint involved cumulatively (46%, 52%, 45%, 38%, respectively) [2,17,22,23,24]. Upper extremity joints were involved in less than 10% of our patients.

Earlier reports demonstrated that juvenile AS had worse functional outcome compared to that of adult AS [25]. Early detection and treatment of axial disease may improve overall disease outcome, especially with biological DMARDs therapy. Sacroiliitis occurred in 61% of our ERA children, of which 40% presented at the time of diagnosis. This latter figure was comparable with reports from China, but much higher than those from other cohorts (16–29%) except that from the US cohort (56%), Table 4. As expected, about one-third of the patients were asymptomatic and contrast-enhanced MRI of the SIJ with restricted modified Schober’s test was done due to their HLA-B27-positive status, as we do not routinely obtain MRI for our patients. The age at the time of first sacroiliitis development was not different between gender, with the median of 13.7 years (*p* = 0.187). The probabilities of developing sacroiliitis were estimated to be 0.3, 0.5 and 0.6 at 0.5, 3 and 5 years after onset, respectively. Knee arthritis at diagnosis (OR 0.237) and corticosteroid use (OR 0.355) were associated with lower risk of sacroiliitis development within the first year of the disease, suggesting that corticosteroids may delay the onset of sacroiliitis. Our patients with early SIJ involvement (at 0.5–1.0 year after onset) seemed to seek medical attention earlier (OR 0.828). Hip arthritis at diagnosis predicted sacroiliitis during the course of the disease (OR 3.941), similar to report by Flato et al. from their Norwegian long-term (median disease duration 15.3 years) ERA cohort, for which hip arthritis within the first 6 months and persistently high ESR increased risk of sacroiliitis [9]. In our previous report on JIA cohort of 251 patients, HLA-B27 (OR 4.943) and older age at onset (OR 1.192) increased the risk of sacroiliitis. When looking at ERA patients only, the gene was not only inversely associated with SIJ involvement (OR 0.311) but also predicted a longer time for sacroiliitis development (HR 0.452), suggesting that there are other non-HLA-B27 genes and/or other factors influencing the axial disease pathogenesis and phenotype of our local ERA population. However, high background of HLA-B27-positive rate or the presence of “disease neutral” HLA-B27 subtype/s (8.9% of HLA-B27*06 subtype in HLA-B27 healthy local population) in our ERA cohort may, in part, have contributed to this discrepancy [26,27]. Further HLA-B27 subtyping and other genetic studies are currently underway. Moreover, female gender, hip arthritis at diagnosis and older age at onset predicted shorter time for sacroiliitis. We did not find that the number of active joints and number of enthesitis increased the risk of sacroiliitis as reported by Pagnini et al. in their Italian ERA cohort [28].

Enthesitis is one of the hallmarks of ERA diagnosis. Our ERA patients had less prevalence of enthesitis both at the time of diagnosis (24%) and along the course of the disease (43%) compared with reports elsewhere [2,5,17,19]. The Achilles tendon insertion site was the most common site (89%), similar to the Chinese cohort (50%) but different from the US cohort where infrapatellar tendon insertion site (44%) and from Canadian cohort where plantar fascial insertion site (39%) was the most common site for enthesitis, suggesting that the predominant enthesitis sites vary among geographical area and biological explanation remains unclear [2,17,29].

### 4.4. Treatment

NSAIDs remained the mainstay first line therapy for our ERA patients, similar to reports elsewhere [2,4,17,23]. The majority of ERA patients had SSZ (79%), as it is our practice to use SSZ in all HLA-B27-positive patients unless G6PD deficiency or intolerable adverse reaction occurred. Methotrexate (MTX, 77%) was the second most common DMARD, and MTX failure was the prerequisite before biologics financial aid locally. Considering only patients with peripheral joint arthritis, MTX failure was estimated to be 63%, which is much less than the efficacy of MTX in other JIA categories [30,31,32]. Furthermore, 85% of patients with sacroiliitis failed MTX, requiring aTNF therapy. Compared with experience from the Taiwanese ERA cohort, they noted 69% MTX refractory rate, which was less than our whole MTX-refractory ERA rate at 78% [4]. Overall, 15.4% of biologics use was reported by Gmuca et al. from their multicentered US ERA cohort, and patients with sacroiliitis were taking NSAIDs only in 40% of cases, biologic monotherapy in 32% and combination of DMARD and biologics in 4%, which was much less than our and other Asian cohorts (Table 4). Intrinsic disease aggressiveness may partly explain this discrepancy. As expected, corticosteroids predicted MTX use (OR 3.101), and MTX predicted aTNF use (OR 3.224). The latter concurred with our high prevalence of MTX failure either for peripheral or axial joint diseases. Furthermore, longer delay in the diagnosis predicted a higher chance of aTNF therapy (OR 1.087), underscoring the “window of opportunity” concept for all JIA treatment.

### 4.5. Outcomes at Last Visit

Similar to the Canadian ReACCh-Out cohort, with a comparable follow-up duration of 4-5 years, disease remained active in one-third of our ERA patients at their last visit [20]. The active disease proportion was much lower than that in the Taiwanese report of 63%, despite a longer follow-up duration (mean 7.7 ± 5.9 years), much less axial disease (16% with sacroiliitis) and higher use of biologics (78%) [4]. It is unclear if their peripheral joint disease was more aggressive or a long delay in biologics initiation contributed to this discrepancy. The opposite could be applied, as only 4.5% of a recent Turkish single-centered ERA cohort had active disease after a follow-up period of 2.8 ± 2.0 years [33]. The latter cohort had 93% of their ERA patients remaining on medication (ROM) after inactive disease was achieved, and this could affect the flare rate, thus decreasing the active disease number. Although two-third of our ERA patients were in non-active disease, only 8% could be totally off all medications, similar to that of Taiwanese (8%) and Turkish (2.3%) cohort studies, but it was significantly less than that of Canadian cohort (52%) despite similar follow-up duration and less biologic use: 22% vs. 60–78% [4,20,33]. This observation likely underscores the impact of genetic and/or environmental factors in treatment response in ERA patients. Similar to other JIA categories reported previously, there was no joint deformity and disability in our cohort [6].

### 4.6. Study Strengths and Limitations

This study represents the first and largest real-life monocentric ERA longitudinal study in the region. The data were collected from the sole and stand-alone children’s hospital in Singapore, where the majority of pediatric rheumatic disease patients are referred. The assessment and treatment patterns were uniform, and the referral bias was minimized, as healthcare was accessible regardless of socioeconomic status and with good support from the local government. Since MTX failure was the prerequisite for biologics financial aid, the real-life effectiveness of MTX in either peripheral joint or axial diseases could be estimated for our ERA patients. There are limitations in this study. First, it was not our practice to examine all 33 entheseal sites routinely, and we did not standardize the entheses examination, thus the number of enthesitis could be underreported [29]. However, we routinely examined the major entheses of the lower extremities where the majority of tender entheseal sites were involved, thus our reported enthesitis rate remained valid [29]. Second, contrast-enhanced MRI of the SIJ was not routinely obtained for every ERA patient unless they were symptomatic or restricted modified Schober’s test was identified. Therefore, the number of axial diseases might have been underestimated. Third, we did not send any microbiological tests routinely unless patients were symptomatic or had preceding history of infections. However, this practice has been performed elsewhere around the world.

## 5. Conclusions

We described the clinical characteristic, treatment and outcomes of an ERA cohort from Singapore, where ERA is the most common category encountered. Similar to reports elsewhere, early adolescent males composed the majority. Almost all patients had peripheral joint disease with oligoarthritis at presentation. More patients with HLA-B27 (82%), while less family history of HLA-B27-related diseases (7.5%) and acute anterior uveitis rate (3.4%), were observed, compared to other cohorts. Sacroiliitis was found in 40% at diagnosis, but enthesitis was less common. Hip arthritis at presentation predicted axial disease. Surprisingly, females and HLA-B27-negative patients carried a higher risk for sacroiliitis, underscoring non-HLA-B27 genetic and/or environmental factors in the immunopathogenesis of ERA locally. This study showed that MTX is less effective in controlling axial disease, as over 80% of sacroiliitis failed MTX therapy. Delay in diagnosis predicted aTNF use. Although two-thirds of our ERA patients successfully achieved non-active disease, <10% could be totally off all medications at the end of the study. Overall functional outcomes were excellent without any disability.

## Figures and Tables

**Table 1:** Patient clinical characteristics and treatment by Human Leukocyte Antigen (HLA)-B27 status.

	Total	HLA–B27 +ve	HLA–B27 −ve	*p* ^±^
Number (%)	146 (100.0)	120 (82.2)	26 (17.8)	
Male, *n* (%)	127 (87.0)	105 (87.5)	22 (84.6)	0.748
Ethnicity, *n* (%)				
Chinese	119 (81.5)	98 (81.7)	21 (80.8)	0.915
Malay	3 (2.1)	2 (1.6)	1 (3.8)	0.447
Indian	7 (4.8)	3 (2.5)	4 (15.4)	0.019
Others	17 (11.6)	17 (14.2)	0	0.043
Age at onset *	11.9 (9.4–14.0)	12.1 (9.5–14.1)	11.0 (9.1–13.2)	0.227
Duration from onset to diagnosis (months) *	2.9 (1.2–7.1)	2.5 (1.1–6.5)	3.5 (1.8–10.3)	0.198
Duration of disease (years) *	4.9 (2.6–8.3)	4.8 (2.6–8.3)	5.0 (3.4–6.6)	0.943
Family history ^†^	11 (7.5)	10 (8.3)	1 (3.8)	0.689
Uveitis, *n* (%)	5 (3.4)	5 (4.2)	0	0.586
Pauci-arthritis at diagnosis	131 (89.7)	107 (89.2)	24 (92.3)	0.632
Active joint counts *	2.0 (1.0–3.0)	2.0 (1.0–3.0)	1.5 (0–2.25)	0.158
Hip arthritis at diagnosis	64 (43.8)	51 (42.5)	13 (50.0)	0.519
Hip arthritis ever	88 (60.3)	70 (58.3)	18 (69.2)	0.379
Sacroiliitis at diagnosis	58 (39.7)	38 (31.7)	20 (76.9)	<0.001
Sacroiliitis ever	89 (61.0)	68 (56.7)	21 (80.8)	0.027
Enthesitis at diagnosis	35 (24.0)	27 (22.5)	8 (30.8)	0.447
Enthesitis ever	62 (42.5)	51 (42.5)	11 (42.3)	0.986
ANA, *n* (%)	19 (13.0)	16 (13.3)	3 (11.5)	0.805
ESR at diagnosis *	32 (14.0–65.0)	34 (15.0–64.8)	15.5 (9.0–77.0)	0.330
CRP at diagnosis *	12.1 (2.5–28.9)	13.6 (4.1–13.1)	7.4 (0.4–22.4)	0.081
Medication, *n* (%)				
NSAIDs	142 (97.3)	116 (96.7)	26 (100.0)	0.345
Systemic steroids	76 (52.1)	65 (54.2)	11 (42.3)	0.272
Intraarticular steroids injection	43 (29.5)	39 (32.5)	4 (15.4)	0.099
Methotrexate	113 (77.4)	90 (75.0)	23 (88.5)	0.196
Sulfasalazine	115 (78.8)	108 (90.0)	7 (26.9)	<0.001
Leflunomide	1 (0.7)	1 (0.8)	0	1.000
Biologics				
Anti-TNF	106 (72.6)	90 (75.0)	16 (61.5)	0.224
Secukinumab	1 (0.7)	1 (0.8)	1 (3.8)	0.325
Ustekinumab	1 (0.7)	1 (0.8)	0	1.000

* median (interquartile range, IQR), ^±^ compared between HLA-B27 positive (+ve) and HLA-B27 negative (−ve) groups, ^†^ History of ankylosing spondylitis, enthesitis-related arthritis, sacroiliitis with inflammatory bowel disease, Reiter’s syndrome, or acute anterior uveitis in a first-degree relative, ESR = Erythrocyte Sedimentation Rate, CRP = C-Reactive Protein.

**Table 2:** ILAR criteria fulfilled at the time of diagnosis (*n* = 146).

ILAR Criteria at Diagnosis	*n* (%)
Arthritis and enthesitis	27 (18.5)
Arthritis only	111 (76.0)
Enthesitis only	8 (5.5)
The presence of or a history of SI tenderness and/or inflammatory lumbosacral pain	58 (39.7)
The presence of HLA-B27 antigen	120 (82.2)
Onset of arthritis in a male over 6 years of age	124 (84.9)
Acute (symptomatic) anterior uveitis	0
History of ankylosing spondylitis, enthesitis-related arthritis, sacroiliitis with inflammatory bowel disease, Reiter’s syndrome, or acute anterior uveitis in a first-degree relative	11 (7.5)

ILAR = International League of Associations for Rheumatology, SI = sacroiliac joint, HLA = Human leukocyte antigen.

**Table 3:** Cumulative probabilities of sacroiliitis development, initiation of MTX or anti-TNF therapy (*n* = 146).

Clinical Parameter/Year	0.5	1	2	3	4	5
Sacroiliitis development	0.274	0.364	0.448	0.498	0.531	0.578
MTX initiation	0.596	0.649	0.716	0.748	0.783	0.814
Anti-TNF therapy initiation	0.281	0.461	0.581	0.674	0.739	0.775

*n* = 146, SI = sacroiliac joint, MTX = methotrexate, TNF = tumor necrosis factor.

**Table 4:** Comparative data on enthesitis-related arthritis (ERA) characteristics across regions.

Country	Singapore	Taiwan	Thailand	India	China	Turkey	France	USA
Number of centers	Single	Single	Single	Single	Single	Multicenter	Single	Multicenter
Year	2020	2019	2015	2015	2015	2011	2018	2017
Number	146	73	39	107	146	120	114	234
Male (%)	87.0	86.3	76.9	91.6	81.5	80.0	63.0	72.2
Age at onset (years)	11.9 (9.4–14.0) *	11.0 ± 3.2 ^†^	10.4 ± 2.8 ^†^	12.0 (4.0–16.0) *	10.3 ^†¶^	15.33 ± 2.9	9.55 (2.7)	11.6 (9.8–13.7) *^¶^
Family history (%)	7.5	11.0	NA	14.0	27.4	NA	28.0	15.4
HLA-B27 positive (%)	82.2	91.8	71.8	79.0	58.9	63.3	43.0	59.2
Uveitis (%)	3.4	9.6	5.1	12.1	7.5	6.7	NA	5.6
Sacroiliitis ^¶^ (%)	39.7	16.4	NA	21.5	43.8	NA	29.0	55.6
Enthesitis ^¶^ (%)	24.0	NA	NA	58.9	37.0	NA	72.0	75.2
Sulfasalazine (%)	78.8	61.6	92.3	NA	NA	100.0	NA	NA
Methotrexate (%)	77.4	74.0	84.6	NA	NA	NA	NA	NA
Biologics (%)	72.6	78.1	56.4	NA	68.5	NA	NA	15.4

* Median (Interquartile range, IQR), ^†^ Mean ± standard deviation (SD), ^±^ at onset, ^¶^ at diagnosis.

## Data Availability

The data presented in this study are available on request from the corresponding author. The data are not publicly available due to ethical reason.

## Supplementary Materials

The following are available online at https://www.mdpi.com/2077-0383/10/4/568/s1, Table S1: Clinical characteristics by presence of sacroiliitis ever, *n* = 146; Table S2: Predictors of clinical variables in our ERA cohort, *n* = 146; Table S3: Predictors of shorter time to develop sacroiliitis in our ERA cohort, *n* = 146.
