# Supplementary material for: Enthesitis Related Arthritis in a Longitudinal Southeast Asian Registry: High Prevalence of HLA-B27, Different Sacroiliitis Risk Factors and Less Common Drug-Free Remission
Source: J Clin Med. 2021 Feb 3;10(4):568. doi: 10.3390/jcm10040568 (PMC7913299; doi:10.3390/jcm10040568)
Supplement: Supplementary file 1 [file jcm-10-00568-s001.pdf]

Supplemental Materials

Supplemental Table 1 Clinical characteristics by presence of sacroiliitis ever, *n* = 146

	Sacroiliitis (*n* = 89)		No sacroiliitis (*n* = 57)		*p*
	n	%	*n*	%	
Male	75	84.3	52	91.2	0.314
Age at onset (year)*	12.2	9.7 – 14.2	11.9	8.9 – 13.8	0.360
Duration from onset to diagnosis*	2.9	1.1 – 7.4	2.9	1.4 – 7.2	0.825
HLA-B27 positive	68	76.4	52	91.2	0.027
Family history	9	10.1	2	3.5	0.202
ESR at diagnosis (mm/hr)*	30	14 – 62	47	14 – 70	0.312
CRP at diagnosis (mg/L)*	11.8	1.6 – 25.4	13.7	5.6 – 35.9	0.151
Hip arthritis ever	66	74.2	22	38.6	<0.001
Enthesitis ever	43	48.3	19	33.3	0.087
Received anti-TNF	77	86.5	29	50.9	<0.001

*Median (interquartile range, IQR)

Supplemental Table 2 Predictors of clinical variables in our ERA cohort, *n* = 146

**Clinical variables**	**Univariate analysis**			**Multivariate analysis**		
	OR	95%CI	*p*	OR	95%CI	*p*
Sacroiliitis developed within 1st 6 months						
Duration from onset to diagnosis	0.737	0.617-0.881	0.001	0.708	0.577-0.869	0.001
Corticosteroids use	0.280	0.116-0.674	0.005	0.212	0.074-0.611	0.004
Sacroiliitis developed within 1st year						
Male	0.201	0.042-0.961	0.044			
Duration from onset to diagnosis	0.860	0.778-0.952	0.004	0.828	0.735-0.932	0.002
Knee arthritis at diagnosis	0.052	0.164-1.007	0.406	0.237	0.080-0.705	0.010
Corticosteroids use	0.371	0.155-0.890	0.026	0.355	0.128-0.982	0.046
Sacroiliitis developed during the course						
HLA-B27	0.311	0.110-0.881	0.028	0.311	0.106-0.913	0.034
Hip arthritis at diagnosis	3.938	1.890-8.206	< 0.001	3.941	1.869-8.311	< 0.001
Ankle arthritis at diagnosis	0.349	0.163-0.745				
Midfoot arthritis at diagnosis	0.141	0.029-0.689				

Subtalar joint arthritis at diagnosis	0.249	0.062-1.007				
<u>Anti-TNF use</u>						
Duration from onset to diagnosis	1.081	0.999-1.170	0.053	1.087	1.005-1.176	0.038
Sacroiliitis at diagnosis	2.100	0.950-4.640	0.067			
ANA positive	3.629	0.799-16.488	0.095			
MTP involvement at diagnosis	0.469	0.196-1.124	0.089			
Methotrexate use	2.933	1.296-6.637	0.010	3.224	1.379-7.539	0.007

ERA = Enthesitis-related arthritis, OR = Odd ratio, CI = Confidence interval, TNF = Tumor necrosis factor, ANA = Antinuclear antibodies,
MTP = Metatarsophalangeal joint

Supplemental Table 3 Predictors of shorter time to develop sacroiliitis in our ERA cohort, *n* = 146

**Clinical variables**	**Univariate analysis**			**Multivariate analysis**		
	HR	95%CI	*p*	HR	95%CI	*p*
Male	0.566	0.319-1.005	0.052	0.508	0.282-0.914	0.024
HLA-B27	0.434	0.264-0.714	0.001	0.452	0.272-0.752	0.002
Older age at onset	1.078	1.002-1.160	0.044	1.076	1.000-1.156	0.049
Hip involvement at diagnosis	2.016	1.325-3.069	0.001	2.128	1.390-3.257	0.001
Knee involvement at diagnosis	0.692	0.444-1.079	0.105			
Ankle involvement at diagnosis	0.470	0.273-0.809	0.006			
MTP involvement at diagnosis	0.584	0.013-0.887	0.085			
